# Comparative Associations of Inflammation and Nutrition-Based Indices with No-Reflow and Mortality After Primary PCI for ST-Elevation Myocardial Infarction

**DOI:** 10.3390/biomedicines14102261

**Published:** 2026-10-06

**Authors:** Ömer Bedir, Tayfur Erdoğdu, Caner Türkoğlu, Remziye Doğan, Şerafettin Demir, İbrahim Halil Tanboğa, Yusuf Ziya Şener

**Affiliations:** 1 Department of Cardiology, Adana City Training and Research Hospital, 01230 Adana, Türkiye; canerturkoglu83@hotmail.com (C.T.); demirkardiyoloji@hotmail.com (Ş.D.); 2 Department of Cardiology, Adana Karşıyaka State Hospital, 01280 Adana, Türkiye; doktortayfur@yahoo.com.tr; 3 Department of Cardiology, Hisar Intercontinental Hospital, 34768 Istanbul, Türkiye; remziye_2148@hotmail.com; 4 Departments of Cardiology and Biostatistics/Bioinformatics, Atlas University Medical School, 34408 Istanbul, Türkiye; haliltanboga@yahoo.com; 5 Department of Internal Medicine, Adana Karşıyaka State Hospital, 01280 Adana, Türkiye; 6 Thoraxcenter, Department of Cardiology, Erasmus MC University Medical Center, 3015 GD Rotterdam, The Netherlands

**Keywords:** percutaneous coronary intervention, ST-elevation myocardial infarction, no-reflow, systemic inflammation, nutritional status, GINI, prognostic nutritional index, neutrophil-to-lymphocyte ratio, systemic immune-inflammation index, all-cause mortality

## Abstract

**Background/Objectives:** Acute myocardial infarction is accompanied by thromboinflammation, immune-cell redistribution and changes in nutritional reserve. We compared six routinely calculable indices under a common modelling strategy in patients with ST-segment elevation myocardial infarction (STEMI) undergoing primary percutaneous coronary intervention (PCI). **Methods:** This retrospective cohort included 2613 patients. The primary endpoint was long-term all-cause mortality, analysed with Cox regression; angiographic no-reflow, in-hospital mortality and 30-day mortality were analysed with logistic regression. The global immune-nutrition-inflammation index (GINI), prognostic nutritional index (PNI), controlling nutritional status (CONUT) score, systemic immune-inflammation index (SII), neutrophil-to-lymphocyte ratio (NLR) and Naples prognostic score (NAPLES) were evaluated in separate models. Continuous indices were modelled flexibly, and every effect estimate compared the 75th with the 25th percentile. **Results:** No-reflow occurred in 353 patients (13.5%), in-hospital death in 102 (3.9%), 30-day death in 157 (6.0%) and long-term death in 466 (17.8%). Median recorded follow-up was 34 [14–46] months. After clinical adjustment, the Q3-versus-Q1 hazard ratios for long-term mortality were 1.17 (95% CI 1.00–1.36) for GINI, 0.79 (0.68–0.91) for PNI, 1.19 (1.01–1.40) for CONUT, 1.20 (1.04–1.39) for SII, 1.24 (1.07–1.44) for NLR and 1.13 (0.98–1.31) for NAPLES. After Holm correction across the six primary tests, PNI, SII and NLR retained evidence of association. In angiographically expanded no-reflow models, GINI and NLR retained Q3-versus-Q1 associations. **Conclusions:** Admission inflammatory and immune-nutrition indices showed outcome-specific prognostic associations. PNI, SII and NLR had the most consistent multiplicity-sensitive long-term associations, whereas GINI and NLR retained associations with no-reflow after angiographic adjustment. These findings do not establish causal effects, predictive superiority or readiness for clinical decision-making.

## 1. Introduction

Primary PCI is the preferred reperfusion strategy for STEMI, yet restoration of epicardial patency does not guarantee tissue-level reperfusion [1]. Distal embolisation, endothelial swelling, platelet–neutrophil interactions, vasoconstriction, reperfusion injury and pre-existing microvascular dysfunction may culminate in microvascular obstruction and angiographic no-reflow [2,3,4,5]. No-reflow is clinically relevant because it is associated with larger infarcts, impaired ventricular recovery, heart failure and mortality [4,5,6].

The host response to acute myocardial injury extends beyond the culprit artery. Neutrophils contribute to capillary plugging, oxidative injury and extracellular-trap formation; monocytes participate in inflammatory expansion and repair; platelets connect thrombosis with innate immunity; and lymphopenia may reflect neurohormonal stress and reduced immune reserve [7,8]. Excessive or unresolved inflammation can amplify endothelial dysfunction, thrombosis, microvascular injury and adverse remodelling across cardiovascular diseases [9,10]. Admission hyperglycaemia may further intensify oxidative stress, inflammation and thrombotic burden, while chronic dysglycaemia and diabetes influence infarct biology and longer-term outcomes through partly distinct pathways [11,12,13,14].

Routinely measured blood variables can be combined into indices that summarise overlapping inflammatory, immune and nutritional domains. The neutrophil-to-lymphocyte ratio (NLR) and the systemic immune-inflammation index (SII) emphasise neutrophil–lymphocyte balance, with SII additionally incorporating platelets [15,16,17,18,19]. The prognostic nutritional index (PNI), the controlling nutritional status (CONUT) score and the Naples prognostic score (NAPLES) combine albumin, lymphocytes, cholesterol and selected cell-count ratios; in the setting of acute infarction they should be interpreted as markers of an inflammatory-metabolic and host-reserve phenotype rather than pure measures of chronic malnutrition [20,21,22,23,24,25,26,27,28,29]. The global immune-nutrition-inflammation index (GINI) integrates C-reactive protein (CRP), neutrophils, monocytes, platelets, albumin and lymphocytes and has recently been evaluated in STEMI [30,31].

Comparisons among these indices are easily distorted by differences in scale, arbitrary thresholds, unequal model specifications and differing availability of component measurements. Moreover, an adjusted association does not establish causal effect, individual prediction accuracy or incremental clinical utility. We therefore compared GINI, PNI, CONUT, SII, NLR and NAPLES using a common prespecified clinical adjustment framework, distribution-based contrasts and flexible modelling of continuous relationships. The primary objective was to quantify their associations with long-term all-cause mortality using time-to-event analysis. Secondary objectives were to examine angiographic no-reflow, in-hospital mortality and 30-day mortality and to evaluate whether the principal associations persisted after endpoint-specific adjustment.

## 2. Materials and Methods

### 2.1. Study Design and Population

**Figure 1:**
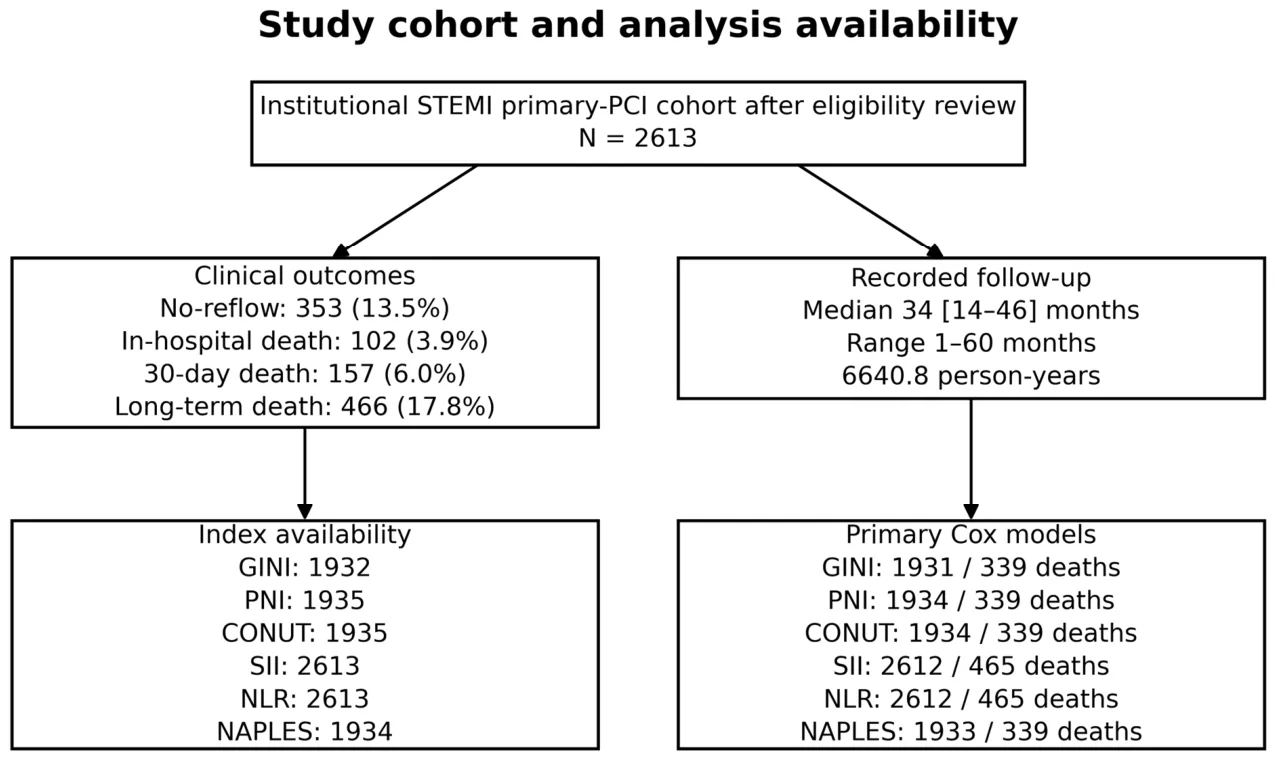
Study cohort and analysis availability. The final institutional cohort comprised 2613 patients. Model-specific complete-case sample sizes are shown. Study-specific eligibility exclusions are described in Section 3.1. The Cox sample counts shown correspond to the primary time-to-event models (Section 3.3); the additional diagnostic refits are reported separately in Supplementary Table S9. Abbreviations: CONUT, controlling nutritional status; GINI, global immune-nutrition-inflammation index; NAPLES, Naples prognostic score; NLR, neutrophil-to-lymphocyte ratio; PCI, percutaneous coronary intervention; PNI, prognostic nutritional index; SII, systemic immune-inflammation index; STEMI, ST-segment elevation myocardial infarction.

We conducted a retrospective observational cohort study at Adana City Training and Research Hospital. Patients evaluated for STEMI between January 2021 and March 2026 were eligible if they presented within 12 h of symptom onset, had an angiographic coronary stenosis greater than 50%, and survived to completion of primary PCI. Patients presenting more than 12 h after symptom onset (*n* = 83), those without a coronary stenosis greater than 50% (*n* = 34), and those in whom primary PCI was not performed or was not completed, including deaths before or during the procedure (*n* = 17), were excluded. The study included all 2613 patients who met these criteria. Procedure completion did not imply normal final coronary flow; patients with angiographic no-reflow remained eligible. Patient selection is summarised in Figure 1. The study protocol was approved by the institutional review board (approval no. 1430; 25 June 2026), and the requirement for written informed consent was waived because of the retrospective design.

Clinical presentation, cardiovascular history, admission laboratory measurements, left ventricular ejection fraction (LVEF), angiographic characteristics and outcomes were obtained from the institutional clinical registry. Laboratory components used for index calculation were obtained during the admission evaluation before primary PCI. The exact interval from blood sampling to wire crossing, whether every component originated from a single specimen and assay-specific handling of values outside detection limits were not retained. The study is reported according to the Strengthening the Reporting of Observational Studies in Epidemiology (STROBE) statement, with relevant marker-reporting principles adapted from the Reporting Recommendations for Tumour Marker Prognostic Studies (REMARK).

### 2.2. Clinical, Angiographic and Glycaemic Variables

Baseline variables included age, sex, diabetes mellitus, hypertension, smoking, dyslipidaemia, previous myocardial infarction, previous PCI, previous coronary artery bypass grafting, peripheral artery disease, four-category Killip class, cardiogenic shock, hypotension, total ischaemic time, renal function, admission glucose, haematologic and inflammatory measurements, Synergy between percutaneous coronary intervention with TAXUS and Cardiac Surgery (SYNTAX) score, multivessel disease, infarct-related artery, pre-PCI Thrombolysis in Myocardial Infarction (TIMI) flow and thrombus burden. LVEF was recorded after PCI. It was therefore excluded from all primary models and entered only in conditional sensitivity analyses. Diabetes status and admission glucose were available, whereas glycated haemoglobin and serial measures of chronic glycaemia were not.

The core adjustment set was chosen before inspection of index-specific results on the basis of timing, clinical relevance and prior prognostic evidence rather than univariable *p*-values. It comprised age, sex, diabetes mellitus, hypertension, four-category Killip class and creatinine. Four-category Killip class was used as the single haemodynamic-severity construct in primary models; cardiogenic shock, hypotension and systolic blood pressure were not entered simultaneously.

### 2.3. Inflammation and Immune-Nutrition Indices

GINI was calculated as [(CRP × monocyte count × platelet count × neutrophil count)/(albumin × lymphocyte count)]/1000. PNI was calculated as 10 × albumin (g/dL) + 5 × lymphocyte count (10^9^/L). SII was calculated as platelet count × neutrophil count/lymphocyte count, and NLR as neutrophil count/lymphocyte count.

CONUT was the sum of the standard albumin, lymphocyte and total-cholesterol component scores. NAPLES assigned one point each for albumin < 4.0 g/dL, total cholesterol ≤ 180 mg/dL, NLR > 2.96 and lymphocyte-to-monocyte ratio ≤ 4.44; the possible range was 0–4. Higher GINI, CONUT, SII, NLR and NAPLES values indicate greater inflammatory or nutritional-risk burden, whereas higher PNI indicates a more favourable profile. No threshold was selected using outcome information.

### 2.4. Outcomes and Follow-Up

The primary endpoint was long-term all-cause mortality. Secondary endpoints were angiographic no-reflow during the index procedure, in-hospital all-cause mortality and 30-day all-cause mortality. Time zero for survival analysis was the index primary PCI. Follow-up ended at death or the last recorded follow-up time. In the original time-to-event analysis reported in Section 3.3, one patient lacked an eligible follow-up duration and was excluded from the time-to-event models.

No-reflow was defined as final TIMI flow grade 0, 1 or 2 after successful stent implantation in the absence of residual mechanical obstruction, including important residual stenosis, dissection, spasm or angiographically visible thrombus [6]. The endpoint was based on the final angiographic assessment documented in the procedural report. Independent core-laboratory rereading, reader blinding, myocardial blush grade, and interobserver agreement were not available. Pre-PCI TIMI flow and thrombus burden were available for sensitivity adjustment, whereas thrombus aspiration, intracoronary vasodilator use, distal embolisation and bailout-intervention fields were not retained.

Mortality status was ascertained through the national death registry and hospital records.

### 2.5. Statistical Analysis

Continuous variables are presented as median [interquartile range], and categorical variables as *n* (%). Outcome-specific baseline comparisons used the Mann–Whitney U test for continuous variables and the Pearson chi-square test or Fisher exact test for categorical variables, as appropriate.

Each index was evaluated in a separate univariable model and in a separate model adjusted for the common clinical set. Long-term mortality was analysed with Cox proportional-hazards regression. No-reflow, in-hospital mortality and 30-day mortality were analysed with logistic regression. Analyses used complete cases for the relevant index and model covariates, with model-specific sample sizes reported in the corresponding tables. The six indices were not entered simultaneously because several share component variables and a mutually adjusted model would answer a narrower, highly collinear question.

GINI, SII and NLR were natural-log transformed because of right-skewness. GINI, PNI, SII and NLR were represented with three-knot restricted cubic splines using the 10th, 50th and 90th percentiles; CONUT and NAPLES were modelled as linear ordinal terms on their native scales. Because CONUT and NAPLES are short bounded integer scores, they were represented by a single linear term in the primary models to preserve parsimony. As a functional-form sensitivity analysis, each score was additionally modelled using a three-knot restricted cubic spline, and the spline specification was compared with the corresponding linear model by likelihood-ratio testing. Age and log creatinine were also modelled with three-knot restricted cubic splines. To permit scale-consistent interpretation, every effect estimate compares the 75th with the 25th percentile. Overall *p*-values are likelihood-ratio tests for the complete index term. The *p*-value for nonlinearity compares the spline model with the corresponding linear-index model. A contrast-specific *p*-value was not additionally reported.

Expanded no-reflow models added total ischaemic time, infarct-related-artery category, pre-PCI TIMI-flow category, high thrombus burden, SYNTAX score and multivessel disease. Expanded early-mortality models added admission glucose, total ischaemic time and previous myocardial infarction. Post-PCI LVEF was added only in separate conditional sensitivity models. Model complexity was summarised by total degrees of freedom and events per parameter. Coefficient stability for in-hospital and 30-day mortality was evaluated using 500 patient-level bootstrap resamples while retaining prespecified transformations and knot locations.

The six overall Cox tests constituted the primary multiplicity family and were adjusted with the Holm procedure. The 18 overall tests across the three secondary logistic endpoints were evaluated with the Benjamini–Hochberg false-discovery-rate procedure. Effect estimates and 95% confidence intervals remained the principal basis for interpretation. All tests were two-sided. Analyses were performed with Python 3.13.5 (Python Software Foundation, Wilmington, DE, USA) using the NumPy, SciPy, statsmodels and matplotlib packages.

Variable-level availability of index components, adjustment covariates, angiographic variables and outcomes was summarised as available and missing counts (Supplementary Table S10A). Patients with and without admission albumin measurements were compared descriptively using standardised differences and the same group-comparison tests; a single omnibus *p*-value was used for each multi-category variable (Supplementary Table S10B).

Additional diagnostic Cox refits used updated follow-up records while retaining the clinical adjustment set and index specifications described above. The proportional-hazards assumption was assessed using score tests based on Schoenfeld residuals with the Kaplan–Meier time transformation, together with inspection of scaled Schoenfeld residual plots. Multi-parameter spline terms were tested jointly, and index-specific and global results were reported. Departures from proportional hazards were addressed in post hoc sensitivity models allowing all clinical coefficients to vary with log(1 + t), where t is the risk-set event time in months. Additional index-by-time interactions were then tested jointly by likelihood-ratio comparison (Supplementary Table S9). These diagnostics concern the additional refits, rather than the original model fits reported in Section 3.3.

Pairwise associations among the six indices were summarised with Spearman rank correlation coefficients in the subset of patients with all six indices available (Supplementary Table S11); these correlations describe the overlap among the indices and were not used for model selection or to rank the indices.

## 3. Results

### 3.1. Study Population and Clinical Outcomes

The study-specific selection counts comprised 2613 included patients and 134 exclusions: presentation more than 12 h after symptom onset (*n* = 83), absence of a coronary stenosis greater than 50% (*n* = 34), and primary PCI not performed or not completed, including deaths before or during the procedure (*n* = 17). Together, these reported inclusion and exclusion counts account for 2747 patients. All patients meeting the eligibility criteria who survived to completion of primary PCI were included; angiographic no-reflow did not preclude inclusion.

The final cohort included 2613 patients (Figure 1). Median age was 62 [54–70] years; 1881 patients (72.0%) were men, 706 (27.0%) had diabetes mellitus, and 1280 (49.0%) had hypertension. Killip class ≥ 2 was present in 415 patients (15.9%), cardiogenic shock in 94 (3.6%) and hypotension in 246 (9.4%). Median total ischaemic time was 133 [76–219] min, admission glucose 109 [86–152] mg/dL, creatinine 0.84 [0.73–0.97] mg/dL, post-PCI LVEF 46 [40–52]% and SYNTAX score 18.6 [11.1–21.1] (Table 1).

No-reflow occurred in 353 patients (13.5%), in-hospital death in 102 (3.9%), 30-day death in 157 (6.0%) and long-term death in 466 (17.8%). Fifty-five patients died after discharge but within 30 days. Recorded follow-up was 34 [14–46] months (range 1–60), corresponding to 6640.8 person-years. Time-to-event information was available for 2612 patients, among whom 465 deaths occurred. Outcome-specific baseline comparisons are presented in Supplementary Tables S1–S4.

### 3.2. Index Distributions and Analysis Samples

Median GINI was 2.18 [0.71–6.00], PNI 45.73 [41.28–50.28], CONUT 2 [0–3], SII 1365 [829–2345], NLR 5.67 [3.69–9.57] and NAPLES 2 [1–2]. SII and NLR were available in all 2613 patients. Indices containing albumin were available in 1932–1935 patients. Score transformations, knot locations and reported contrasts are shown in Supplementary Table S5A,B.

Albumin was available in 1935 patients and unavailable in 678 (25.9%); variable-level availability is reported in Supplementary Table S10A. Compared with patients without albumin measurements, those with measurements had more dyslipidaemia (40.4% vs. 27.3%; absolute standardised difference 0.280) and less high thrombus burden (81.1% vs. 86.4%; absolute standardised difference 0.144). Long-term death proportions were 17.6% and 18.6%, respectively. The full descriptive comparison is provided in Supplementary Table S10B.

Pairwise Spearman correlations among the six indices are presented in Supplementary Table S11 (common complete sample, *n* = 1932). The strongest correlations were observed between SII and NLR (ρ = 0.927) and between PNI and CONUT (ρ = −0.794), consistent with substantial overlap in their component variables.

### 3.3. Long-Term All-Cause Mortality

In univariable Cox models, each index was associated with long-term mortality (Table 2). After adjustment, higher PNI was associated with a lower hazard of death (HR 0.79, 95% CI 0.68–0.91; overall *p* = 0.001), whereas higher CONUT (HR 1.19, 95% CI 1.01–1.40; *p* = 0.042), SII (HR 1.20, 95% CI 1.04–1.39; *p* = 0.008) and NLR (HR 1.24, 95% CI 1.07–1.44; *p* = 0.002) were associated with a higher hazard. The estimates for GINI (HR 1.17, 95% CI 1.00–1.36; overall *p* = 0.113) and NAPLES (HR 1.13, 95% CI 0.98–1.31; *p* = 0.092) were less precise and included the null. PNI showed evidence of nonlinearity (*p* = 0.026); its Q3-versus-Q1 HR therefore summarises the central contrast rather than the entire fitted relationship.

**Figure 2:**
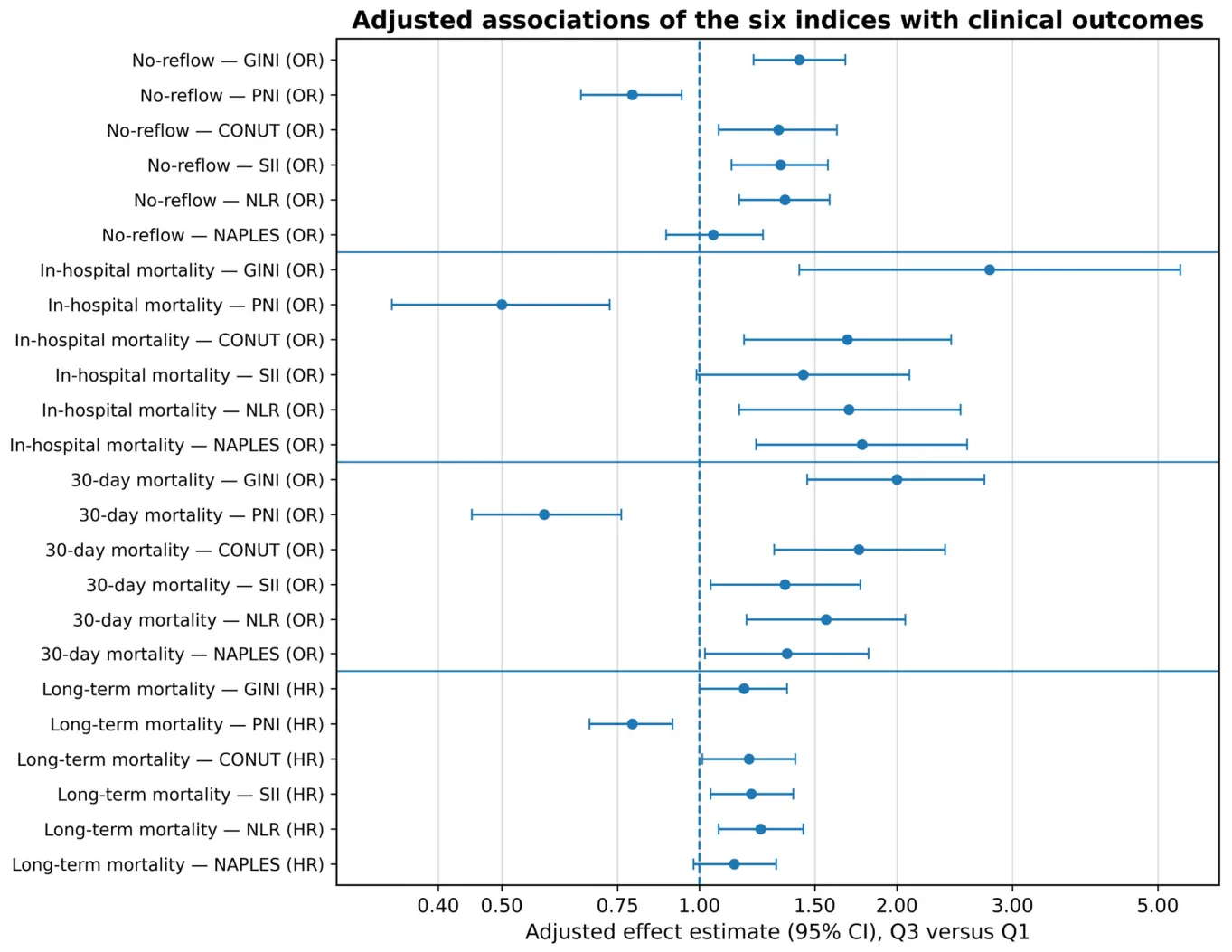
Adjusted associations of six inflammation and immune-nutrition indices with clinical outcomes. Points show adjusted odds ratios for no-reflow, in-hospital mortality and 30-day mortality and adjusted hazard ratios for long-term mortality; bars show 95% confidence intervals. Every estimate compares the 75th with the 25th percentile. Primary models included age, sex, diabetes mellitus, hypertension, four-category Killip class and log creatinine. Post-PCI LVEF was excluded. Abbreviations: CI, confidence interval; CONUT, controlling nutritional status; GINI, global immune-nutrition-inflammation index; HR, hazard ratio; LVEF, left ventricular ejection fraction; NAPLES, Naples prognostic score; NLR, neutrophil-to-lymphocyte ratio; OR, odds ratio; PCI, percutaneous coronary intervention; PNI, prognostic nutritional index; Q1, first quartile (25th percentile); Q3, third quartile (75th percentile); SII, systemic immune-inflammation index.

After Holm correction across the six primary overall tests, adjusted *p*-values were 0.0068 for PNI, 0.0104 for NLR, 0.0309 for SII, 0.1275 for CONUT and 0.1844 for both GINI and NAPLES (Supplementary Table S7). Thus, the multiplicity-sensitive pattern was most consistent for PNI, NLR and SII. Adjusted long-term estimates are displayed with the secondary endpoint estimates in Figure 2.

### 3.4. Angiographic No-Reflow

In primary adjusted models, GINI, PNI, CONUT, SII and NLR showed overall associations with no-reflow, whereas NAPLES did not (Table 3). The Q3-versus-Q1 odds ratios were 1.42 (95% CI 1.21–1.67) for GINI, 0.79 (0.66–0.94) for PNI, 1.32 (1.07–1.62) for CONUT, 1.33 (1.12–1.57) for SII and 1.35 (1.15–1.58) for NLR. GINI showed evidence of nonlinearity (*p* = 0.005).

**Figure 3:**
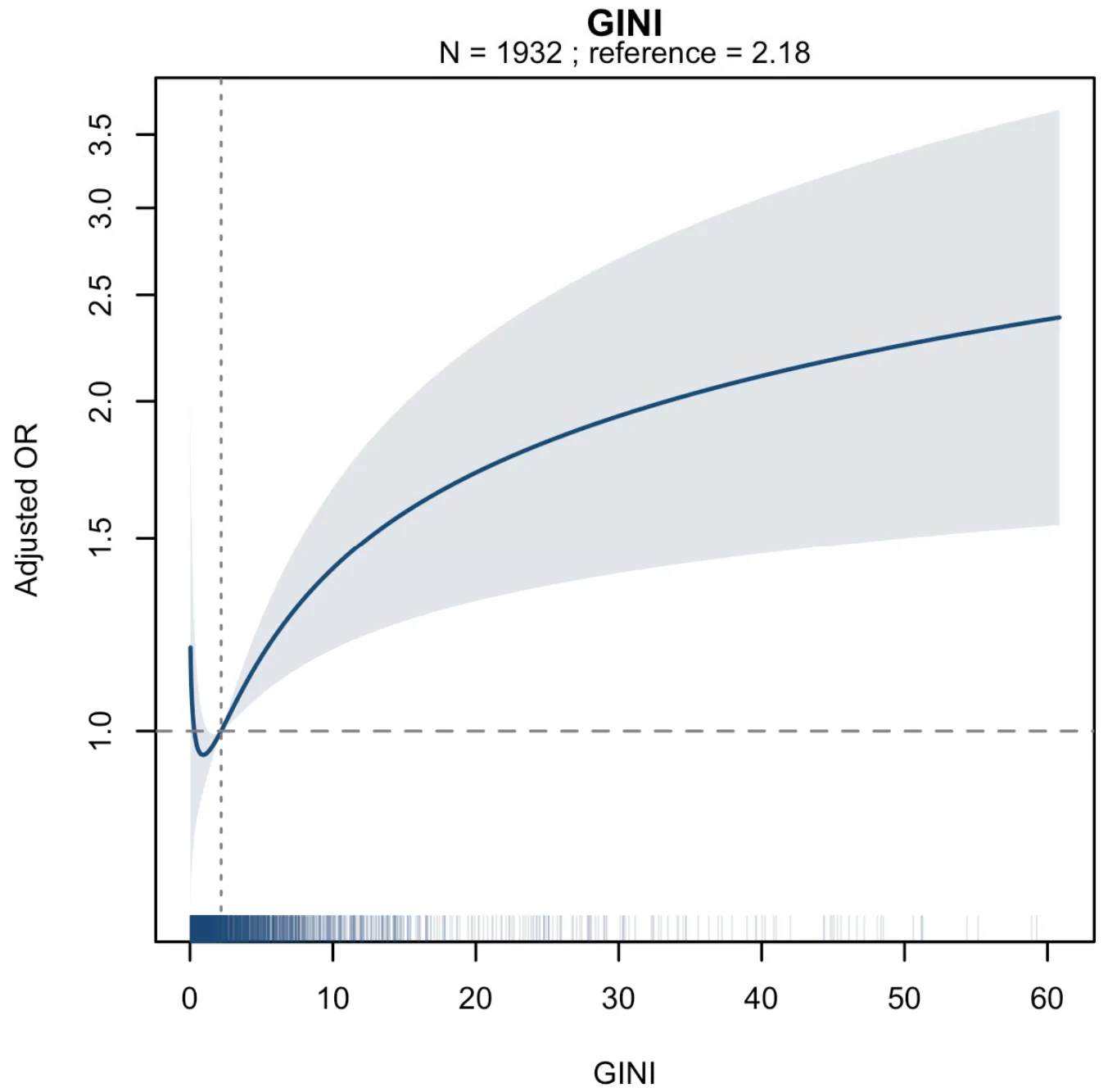
Expanded angiographic no-reflow partial-effect curve for GINI. The curve is from the multivariable model including the core clinical variables, total ischaemic time, infarct-related artery, pre-PCI TIMI flow, high thrombus burden, SYNTAX score and multivessel disease. The median value is the reference; the band is the pointwise 95% confidence interval, and the rug shows the observed distribution. Abbreviations: GINI, global immune-nutrition-inflammation index; PCI, percutaneous coronary intervention; SYNTAX, Synergy between Percutaneous Coronary Intervention with TAXUS and Cardiac Surgery; TIMI, Thrombolysis in Myocardial Infarction.

After additional adjustment for total ischaemic time, infarct-related artery, pre-PCI TIMI flow, high thrombus burden, SYNTAX score and multivessel disease, the central contrasts remained most clearly estimated for GINI (OR 1.28, 95% CI 1.08–1.52) and NLR (OR 1.19, 95% CI 1.01–1.41). SII retained an overall index-term association, although its Q3-versus-Q1 interval included 1.0 (OR 1.17, 95% CI 0.99–1.39), indicating that the fitted relationship was not fully represented by one central contrast (Table 4 and Figure 3).

### 3.5. In-Hospital and 30-Day Mortality

For in-hospital mortality, the adjusted Q3-versus-Q1 odds ratios were 2.77 (95% CI 1.42–5.41) for GINI, 0.50 (0.34–0.73) for PNI, 1.68 (1.17–2.42) for CONUT, 1.44 (0.99–2.09) for SII, 1.69 (1.15–2.50) for NLR and 1.77 (1.22–2.56) for NAPLES (Table 5A). The SII central contrast was imprecise despite an overall index-term *p*-value of 0.015. The smaller event count produced wider intervals than for the other endpoints.

For 30-day mortality, all six indices showed adjusted overall associations. The corresponding Q3-versus-Q1 odds ratios were 2.00 (95% CI 1.46–2.72) for GINI, 0.58 (0.45–0.76) for PNI, 1.75 (1.30–2.37) for CONUT, 1.35 (1.04–1.76) for SII, 1.56 (1.18–2.06) for NLR and 1.36 (1.02–1.81) for NAPLES (Table 5B). The 55 deaths occurring after discharge but within 30 days demonstrate that the 30-day endpoint captured a clinically relevant early post-discharge period beyond the index hospitalisation.

### 3.6. Sensitivity, Multiplicity and Stability Analyses

Flexible sensitivity analyses for CONUT and NAPLES did not provide strong evidence of departure from the primary linear ordinal specification. Across the four endpoints, likelihood-ratio *p*-values for nonlinearity ranged from 0.159 to 0.822 for CONUT and from 0.098 to 0.750 for NAPLES (Supplementary Table S12). Expanded early-mortality models that included admission glucose, total ischaemic time and previous myocardial infarction preserved the direction of the principal associations (Supplementary Table S6). Conditional models that added post-PCI LVEF attenuated several estimates, particularly for no-reflow and in-hospital mortality.

Benjamini–Hochberg adjustment across the 18 secondary overall tests preserved the principal no-reflow and early-mortality signals except for NAPLES with no-reflow (Supplementary Table S7). Primary logistic models used 11–12 degrees of freedom; expanded no-reflow models used 23–24 degrees of freedom. Bootstrap analyses completed 499–500 of 500 resamples per early-mortality model. The direction of the Q3-versus-Q1 estimate was retained in 96.2–100% of resamples, although the distributions remained broad for in-hospital mortality, reflecting the limited number of events (Supplementary Table S8).

In the additional Cox refits using updated follow-up records, the overall diagnostic population comprised 2613 patients with 466 deaths; index-specific sample sizes are reported in Supplementary Table S9A. Global proportional-hazards tests indicated departures in all six models (all *p* < 0.001). Index-specific *p*-values were 0.038 for GINI, 0.726 for PNI, 0.692 for CONUT, 0.361 for SII, 0.250 for NLR and 0.915 for NAPLES. After allowing clinical coefficients to vary with time, adding index-by-time terms provided no clear evidence of further improvement (likelihood-ratio *p* = 0.195–0.861; Supplementary Table S9B). Covariate-specific diagnostics are provided in Supplementary Table S9C. These supplementary findings should not be interpreted as demonstrating proportional hazards in the original Table 2 fits.

## 4. Discussion

This study provides four main findings. First, time-to-event analysis identified adjusted long-term associations for PNI, CONUT, SII and NLR, while the GINI and NAPLES intervals included the null. After multiplicity correction across the six primary tests, the pattern was most consistent for PNI, NLR and SII. Second, several indices were associated with no-reflow in the common clinical model, but only GINI and NLR retained clearly estimated central contrasts after additional angiographic adjustment; SII retained a curve-level association with an imprecise interquartile contrast. Third, all six indices were associated with 30-day mortality in the primary adjusted models, whereas in-hospital estimates were less precise because of the smaller event count. Fourth, the attenuation after post-PCI LVEF adjustment supports the interpretation that these indices partly capture acute infarct severity and early ventricular injury rather than isolated inflammatory or nutritional biology.

The results reinforce the concept that acute myocardial infarction is both a focal thrombotic event and a systemic inflammatory-metabolic stress state [9,10]. Neutrophil activation, platelet–leukocyte interactions, acute-phase signalling, lymphocyte depletion and lower albumin concentration may reflect overlapping dimensions of microvascular injury, infarct magnitude, neurohormonal stress, comorbidity and host reserve. The indices are therefore not interchangeable, but neither should they be interpreted as pure measurements of inflammation or malnutrition. Their components are influenced by the timing and severity of acute illness, baseline frailty, renal function, hepatic protein synthesis, vascular permeability and chronic metabolic disease.

The long-term pattern was biologically coherent but more selective than the early-mortality pattern. Higher NLR and SII were associated with higher mortality hazard, consistent with the role of neutrophils and platelets in thromboinflammation and with prior meta-analyses after acute coronary syndromes and PCI [15,16,17,18,19]. Higher PNI was associated with lower hazard, although the nonlinear test indicates that one HR cannot fully describe its functional form. CONUT showed a modest nominal association that did not persist after Holm correction. GINI, despite its dense combination of inflammatory and nutritional components, had an adjusted interval that crossed the null, suggesting that greater compositional complexity does not necessarily translate into a more robust conditional association. NAPLES was similarly imprecise. These findings argue against ranking indices by unadjusted magnitude or by the smallest *p*-value.

The no-reflow analysis provides a complementary message. No-reflow is strongly influenced by ischaemic duration, baseline flow, thrombus burden, lesion and vessel anatomy, distal embolisation, procedural manipulation and microvascular susceptibility [2,3,4,5]. GINI and NLR retained Q3-versus-Q1 associations after adjustment for the major available angiographic factors, while the other central contrasts attenuated. This does not establish a no-reflow-specific mechanism. Rather, it suggests that neutrophil-weighted systemic inflammation and the broader GINI phenotype may capture information not fully represented by the measured angiographic variables. The lack of blinded central adjudication and the absence of several treatment variables preclude stronger interpretation.

The distinction between in-hospital and 30-day mortality is clinically meaningful. The former is influenced by length of stay and captures only the index admission. Thirty-day mortality additionally includes the vulnerable early post-discharge period; in this cohort, 55 deaths occurred after discharge but within 30 days. The broad early-mortality associations are compatible with these indices acting as summary markers of initial haemodynamic compromise, infarct burden, renal dysfunction and host reserve. Their association with mortality should not be interpreted as evidence that modifying the indices themselves would improve outcomes.

Excessive or unresolved inflammation contributes to endothelial dysfunction, plaque instability, thrombosis and adverse remodelling across cardiovascular disease [10]. Hyperglycaemia may intensify thrombus burden and inflammatory activation during STEMI, and prior studies have linked hyperglycaemic states, diabetes and chronic metabolic dysregulation with adverse post-infarction outcomes [12,13,14]. Admission glucose was included in expanded endpoint-specific models, which broadly preserved the principal associations. HbA1c and serial glucose measurements were unavailable, so the study cannot distinguish acute stress hyperglycaemia from chronic hyperglycaemia or test their interaction with the six indices.

Recent comparative reports of immune-inflammatory and nutritional indices in acute coronary syndrome populations similarly show that several correlated scores can be associated with outcome [32,33]. The present study adds a common modelling scale, formal nonlinearity tests, endpoint-specific adjustment and time-to-event analysis. It does not, however, demonstrate comparative predictive superiority. A statistically stronger association does not imply better individual prediction, calibration, clinical utility or transportability. Those questions require a clearly specified clinical use, comparison with established scores such as GRACE or TIMI, optimism-corrected calibration, decision analysis and external validation.

The clearest clinical message is therefore cautious. These low-cost admission indices may identify overlapping high-risk inflammatory and host-reserve phenotypes and may be useful for research-oriented risk enrichment. They should not be used as isolated treatment thresholds, substitutes for established STEMI assessment or validated bedside prediction tools. Future multicentre studies should harmonise blood-sampling times, retain exact event and censoring dates, use independent no-reflow adjudication, compare against established risk models and determine whether any index adds clinically useful information beyond its individual components.

### Strengths and Limitations

Strengths include a large STEMI cohort, six prespecified routinely calculable indices, common Q3-versus-Q1 contrasts, flexible modelling of continuous indices, separate overall and nonlinearity tests, endpoint-specific sensitivity models, multiplicity-sensitive analyses and bootstrap coefficient-stability assessment. The separation of primary baseline models from conditional post-PCI LVEF models also reduces the risk of treating a downstream severity marker as a baseline confounder.

Several limitations are important. The study was retrospective and single-centre, and residual confounding and measurement heterogeneity remain possible. Albumin was unavailable in some patients; albumin-containing indices therefore relied on complete-case samples, which may differ from the full cohort. The six indices were measured once at admission, and serial values were unavailable. No-reflow was based on operator-documented TIMI flow without blinded core-laboratory review or interobserver assessment, and several procedure-response variables were unavailable. Post-PCI LVEF may reflect infarct severity and was therefore restricted to sensitivity analysis. Finally, correlated indices and multiple endpoints were assessed; multiplicity procedures reduce but do not remove the risk of chance findings, and no external validation was performed.

Differences between patients with and without albumin measurements mean that selection bias in the complete-case analyses cannot be excluded. The additional proportional-hazards diagnostics used updated follow-up recorded in months and were reported separately from the original primary estimates; their findings cannot be transferred directly to the original model fits. The time-varying-coefficient models were post hoc and evaluated a specified temporal form, rather than excluding every possible form of time dependence.

## 5. Conclusions

Among patients with STEMI undergoing primary PCI, higher SII and NLR and lower PNI were associated with long-term all-cause mortality after common clinical adjustment and remained supported after correction across the six primary tests. CONUT showed a more modest nominal long-term association, whereas the adjusted GINI and NAPLES intervals included the null. For angiographic no-reflow, GINI and NLR retained Q3-versus-Q1 associations after additional angiographic adjustment. These findings describe conditional prognostic associations and should not be interpreted as causal effects, proof of predictive superiority or support for immediate clinical thresholds. Prospective multicentre studies with exact event times, independent endpoint adjudication and external prediction-model evaluation are required.

## Figures and Tables

**Table 1:** Baseline characteristics and clinical outcomes of the study population.

Characteristic	Overall
Age, years	62 [54–70]
Male sex	1881 (72.0)
Diabetes mellitus	706 (27.0)
Hypertension	1280 (49.0)
Current smoking	1176 (45.0)
Dyslipidaemia	967 (37.0)
Prior myocardial infarction	340 (13.0)
Prior PCI	366 (14.0)
Prior CABG	78 (3.0)
Peripheral artery disease	183 (7.0)
Killip class ≥ 2	415 (15.9)
Cardiogenic shock	94 (3.6)
Hypotension	246 (9.4)
Systolic blood pressure, mmHg	136 [123–147]
Heart rate, beats/min	84 [75–93]
Total ischaemic time, min	133 [76–219]
Creatinine, mg/dL	0.84 [0.73–0.97]
Admission glucose, mg/dL	109 [86–152]
Haemoglobin, g/dL	14.0 [12.7–14.9]
White blood cell count, 10^9^/L	11.7 [9.6–13.9]
Neutrophil count, 10^9^/L	8.8 [6.9–10.8]
Lymphocyte count, 10^9^/L	1.5 [1.0–2.1]
Platelet count, 10^9^/L	238 [200–284]
C-reactive protein, mg/L	7.4 [2.9–13.1]
Albumin, g/dL	3.8 [3.6–4.1]
Total cholesterol, mg/dL	192 [166–213]
Post-PCI LVEF, %	46 [40–52]
SYNTAX score	18.6 [11.1–21.1]
Multivessel coronary disease	1100 (42.1)
High thrombus burden	2156 (82.5)
No-reflow	353 (13.5)
In-hospital mortality	102 (3.9)
30-day mortality	157 (6.0)
Long-term mortality	466 (17.8)
Follow-up, months	34 [14–46]

Continuous variables are median [interquartile range]; categorical variables are *n* (%). Abbreviations: CABG, coronary artery bypass grafting; LVEF, left ventricular ejection fraction; PCI, percutaneous coronary intervention; SYNTAX, Synergy between Percutaneous Coronary Intervention with TAXUS and Cardiac Surgery.

**Table 2:** Univariable and adjusted Cox associations with long-term all-cause mortality.

Index	Contrast (Q3 vs. Q1)	Univariable HR (95% CI)	Univariable Overall *p*	Univariable *p* for Nonlinearity	Adjusted HR (95% CI)	Adjusted Overall *p*	Adjusted *p* for Nonlinearity	N/Deaths
GINI	6.00 vs. 0.71	1.61 (1.38–1.87)	<0.001	0.676	1.17 (1.00–1.36)	0.113	0.976	1931/339
PNI	50.28 vs. 41.28	0.62 (0.54–0.70)	<0.001	<0.001	0.79 (0.68–0.91)	0.001	0.026	1934/339
CONUT	3 vs. 0	1.73 (1.50–2.00)	<0.001	—	1.19 (1.01–1.40)	0.042	—	1934/339
SII	2345 vs. 829	1.48 (1.27–1.71)	<0.001	0.173	1.20 (1.04–1.39)	0.008	0.585	2612/465
NLR	9.57 vs. 3.69	1.54 (1.33–1.79)	<0.001	0.034	1.24 (1.07–1.44)	0.002	0.404	2612/465
NAPLES	2 vs. 1	1.34 (1.17–1.54)	<0.001	—	1.13 (0.98–1.31)	0.092	—	1933/339

All effects compare the 75th with the 25th percentile. Adjusted models included age, sex, diabetes mellitus, hypertension, four-category Killip class, and log creatinine. Continuous clinical covariates and GINI, PNI, SII and NLR were modelled using three-knot restricted cubic splines. Overall *p* tests the complete index term; *p* for nonlinearity tests departure from linearity. Post-PCI LVEF was excluded. The full cohort comprised 2613 patients with 466 recorded long-term deaths. The original time-to-event analysis underlying this table included up to 2612 patients and 465 deaths because one patient with a recorded death lacked an eligible follow-up duration in that analysis. Further reductions reflect index-specific measurement availability. Supplementary Table S9 instead reports additional diagnostic refits using updated follow-up records, with a maximum of 2613 patients and 466 deaths; those diagnostics are not tests of the original fits in this table. Abbreviations: CI, confidence interval; HR, hazard ratio; LVEF, left ventricular ejection fraction; N/deaths, number of patients in the model and number of deaths; PCI, percutaneous coronary intervention; Q1, first quartile (25th percentile); Q3, third quartile (75th percentile); CONUT, controlling nutritional status; GINI, global immune-nutrition-inflammation index; NAPLES, Naples prognostic score; NLR, neutrophil-to-lymphocyte ratio; PNI, prognostic nutritional index; SII, systemic immune-inflammation index.

**Table 3:** Univariable and adjusted associations with angiographic no-reflow.

Index	Contrast (Q3 vs. Q1)	Univariable OR (95% CI)	Overall *p*	*p* for Nonlinearity	Adjusted OR (95% CI)	Overall *p*	*p* for Nonlinearity	N/Events
GINI	6.00 vs. 0.71	1.56 (1.34–1.82)	<0.001	0.002	1.42 (1.21–1.67)	<0.001	0.005	1932/268
PNI	50.28 vs. 41.28	0.64 (0.55–0.75)	<0.001	0.002	0.79 (0.66–0.94)	0.018	0.050	1935/268
CONUT	3 vs. 0	1.68 (1.40–2.01)	<0.001	—	1.32 (1.07–1.62)	0.010	—	1935/268
SII	2345 vs. 829	1.48 (1.26–1.74)	<0.001	0.614	1.33 (1.12–1.57)	<0.001	0.652	2613/353
NLR	9.57 vs. 3.69	1.52 (1.30–1.78)	<0.001	0.385	1.35 (1.15–1.58)	<0.001	0.310	2613/353
NAPLES	2 vs. 1	1.23 (1.05–1.45)	0.010	—	1.05 (0.89–1.25)	0.567	—	1934/268

All effects compare the 75th with the 25th percentile. Adjusted models included age, sex, diabetes mellitus, hypertension, four-category Killip class, and log creatinine. Post-PCI LVEF was excluded. Overall *p* tests the complete index term; *p* for nonlinearity tests departure from linearity. Abbreviations: CI, confidence interval; LVEF, left ventricular ejection fraction; N/events, number of patients in the model and number of events; OR, odds ratio; PCI, percutaneous coronary intervention; Q1, first quartile (25th percentile); Q3, third quartile (75th percentile); CONUT, controlling nutritional status; GINI, global immune-nutrition-inflammation index; NAPLES, Naples prognostic score; NLR, neutrophil-to-lymphocyte ratio; PNI, prognostic nutritional index; SII, systemic immune-inflammation index.

**Table 4:** Expanded angiographic no-reflow models.

Endpoint	Index	Contrast (Q3 vs. Q1)	N/Events	Adjusted Effect (95% CI)	Overall *p*	*p* for Nonlinearity	Model df	Events/Parameter
No-reflow	GINI	6.00 vs. 0.71	1932/268	1.28 (1.08–1.52)	<0.001	0.006	24	11.2
No-reflow	PNI	50.28 vs. 41.28	1935/268	0.84 (0.70–1.01)	0.121	0.120	24	11.2
No-reflow	CONUT	3 vs. 0	1935/268	1.20 (0.97–1.50)	0.095	—	23	11.7
No-reflow	SII	2345 vs. 829	2613/353	1.17 (0.99–1.39)	0.007	0.232	24	14.7
No-reflow	NLR	9.57 vs. 3.69	2613/353	1.19 (1.01–1.41)	<0.001	0.064	24	14.7
No-reflow	NAPLES	2 vs. 1	1934/268	0.99 (0.83–1.18)	0.884	—	23	11.7

Expanded models included the core clinical variables plus total ischaemic time, infarct-related artery, pre-PCI TIMI flow, high thrombus burden, SYNTAX score and multivessel disease. Abbreviations: CI, confidence interval; df, degrees of freedom; N/events, number of patients in the model and number of events; PCI, percutaneous coronary intervention; Q1, first quartile (25th percentile); Q3, third quartile (75th percentile); SYNTAX, Synergy between Percutaneous Coronary Intervention with TAXUS and Cardiac Surgery; TIMI, Thrombolysis in Myocardial Infarction; CONUT, controlling nutritional status; GINI, global immune-nutrition-inflammation index; NAPLES, Naples prognostic score; NLR, neutrophil-to-lymphocyte ratio; PNI, prognostic nutritional index; SII, systemic immune-inflammation index.

**Table 5:** Univariable and adjusted associations with early mortality. Panel (**A**), in-hospital mortality; Panel (**B**), 30-day mortality.

Index	Contrast (Q3 vs. Q1)	Univariable OR (95% CI)	Overall *p*	*p* for Nonlinearity	Adjusted OR (95% CI)	Overall *p*	*p* for Nonlinearity	N/Events
**Panel (A). In-hospital mortality**								
GINI	6.00 vs. 0.71	4.79 (2.41–9.56)	<0.001	0.518	2.77 (1.42–5.41)	<0.001	0.540	1932/75
PNI	50.28 vs. 41.28	0.37 (0.26–0.52)	<0.001	0.094	0.50 (0.34–0.73)	<0.001	0.527	1935/75
CONUT	3 vs. 0	3.00 (2.24–4.02)	<0.001	—	1.68 (1.17–2.42)	0.005	—	1935/75
SII	2345 vs. 829	1.85 (1.33–2.57)	<0.001	0.708	1.44 (0.99–2.09)	0.015	0.979	2613/102
NLR	9.57 vs. 3.69	2.20 (1.53–3.16)	<0.001	0.280	1.69 (1.15–2.50)	0.007	0.250	2613/102
NAPLES	2 vs. 1	2.30 (1.65–3.21)	<0.001	—	1.77 (1.22–2.56)	0.002	—	1934/75
**Panel (B). 30-day mortality**								
GINI	6.00 vs. 0.71	2.80 (2.03–3.86)	<0.001	0.097	2.00 (1.46–2.72)	<0.001	0.155	1932/112
PNI	50.28 vs. 41.28	0.44 (0.36–0.55)	<0.001	<0.001	0.58 (0.45–0.76)	<0.001	0.176	1935/112
CONUT	3 vs. 0	2.77 (2.16–3.56)	<0.001	—	1.75 (1.30–2.37)	<0.001	—	1935/112
SII	2345 vs. 829	1.64 (1.29–2.08)	<0.001	0.072	1.35 (1.04–1.76)	<0.001	0.108	2613/157
NLR	9.57 vs. 3.69	1.87 (1.45–2.42)	<0.001	0.869	1.56 (1.18–2.06)	<0.001	0.880	2613/157
NAPLES	2 vs. 1	1.72 (1.34–2.22)	<0.001	—	1.36 (1.02–1.81)	0.034	—	1934/112

All effects compare the 75th with the 25th percentile. Adjusted models included age, sex, diabetes mellitus, hypertension, four-category Killip class, and log creatinine. Overall *p* tests the complete index term; *p* for nonlinearity tests departure from linearity. Abbreviations: CI, confidence interval; N/events, number of patients in the model and number of events; OR, odds ratio; Q1, first quartile (25th percentile); Q3, third quartile (75th percentile); CONUT, controlling nutritional status; GINI, global immune-nutrition-inflammation index; NAPLES, Naples prognostic score; NLR, neutrophil-to-lymphocyte ratio; PNI, prognostic nutritional index; SII, systemic immune-inflammation index.

## Data Availability

The data are not publicly available owing to privacy and ethical restrictions but may be available from the corresponding author subject to institutional approval.

## Supplementary Materials

The following supporting information can be downloaded at: https://www.mdpi.com/article/10.3390/biomedicines14102261/s1, Table S1: Baseline characteristics according to no-reflow status. Table S2: Baseline characteristics according to in-hospital mortality. Table S3: Baseline characteristics according to 30-day mortality. Table S4: Baseline characteristics according to long-term mortality status. Table S5A: Score distributions, transformations and knot locations. Table S5B: Knot locations for continuous clinical covariates. Table S6: Expanded early-mortality models. Table S7: Multiplicity-sensitive analyses. Table S8: Bootstrap coefficient-stability analysis. Table S9: Proportional-hazards diagnostics and time-varying-coefficient sensitivity analyses in additional Cox refits. Table S10: Variable-level availability and comparison according to albumin availability. Table S11: Spearman correlation matrix among the six indices. Table S12: Linear versus flexible (restricted cubic spline) specifications for CONUT and NAPLES.

## Abbreviations

The following abbreviations are used in this manuscript:

CABG	coronary artery bypass grafting
CI	confidence interval
CONUT	controlling nutritional status
CRP	C-reactive protein
GINI	global immune-nutrition-inflammation index
HR	hazard ratio
IQR	interquartile range
LVEF	left ventricular ejection fraction
NAPLES	Naples prognostic score
NLR	neutrophil-to-lymphocyte ratio
OR	odds ratio
PCI	Percutaneous Coronary Intervention
PNI	prognostic nutritional index
Q1	first quartile (25th percentile)
Q3	third quartile (75th percentile)
REMARK	Reporting Recommendations for Tumour Marker Prognostic Studies
SII	systemic immune-inflammation index
STEMI	ST-segment elevation myocardial infarction
STROBE	Strengthening the Reporting of Observational Studies in Epidemiology
SYNTAX	Synergy between Percutaneous Coronary Intervention with TAXUS and Cardiac Surgery
TIMI	Thrombolysis in Myocardial Infarction

## References

[1] Byrne R.A., Rossello X., Coughlan J.J., Barbato E., Berry C., Chieffo A., Claeys M.J., Dan G.A., Dweck M.R., Galbraith M. (2023). 2023 ESC Guidelines for the management of acute coronary syndromes. Eur. Heart J..

[2] Maino A., Walsh J.L., Portolan L., Kotronias R., Ameri P., Vergallo R., Porto I., Crea F., Banning A., Tammaro P. (2026). Therapeutic interventions for coronary microvascular obstruction after acute myocardial infarction: Developments and challenges. Pharmacol. Ther..

[3] Borzuta H., Kociemba W., Bochenek O., Jarowicz M., Wsół A. (2025). Advances in Pathophysiology and Novel Therapeutic Strategies for Coronary No-Reflow Phenomenon. Biomedicines.

[4] Niccoli G., Scalone G., Lerman A., Crea F. (2016). Coronary microvascular obstruction in acute myocardial infarction. Eur. Heart J..

[5] Jaffe R., Charron T., Puley G., Dick A., Strauss B.H. (2008). Microvascular obstruction and the no-reflow phenomenon after percutaneous coronary intervention. Circulation.

[6] Ndrepepa G., Tiroch K., Fusaro M., Keta D., Seyfarth M., Byrne R.A., Pache J., Alger P., Mehilli J., Schömig A. (2010). 5-year prognostic value of no-reflow phenomenon after percutaneous coronary intervention in patients with acute myocardial infarction. J. Am. Coll. Cardiol..

[7] Hilgendorf I., Frantz S., Frangogiannis N.G. (2024). Repair of the infarcted heart: Cellular effectors, molecular mechanisms and therapeutic opportunities. Circ. Res..

[8] Shirakawa K., Sano M. (2022). Neutrophils and neutrophil extracellular traps in cardiovascular disease: An overview and potential therapeutic approaches. Biomedicines.

[9] Imbesi A., Greco A., Spagnolo M., Laudani C., Raffo C., Finocchiaro S., Mazzone P.M., Landolina D., Mauro M.S., Cutore L. (2025). Targeting inflammation after acute myocardial infarction. J. Am. Coll. Cardiol..

[10] Sardu C., Paolisso G., Marfella R. (2020). Inflammatory related cardiovascular diseases: From molecular mechanisms to therapeutic targets. Curr. Pharm. Des..

[11] Bedir Ö., Polat İ. (2026). Stress hyperglycemia ratio outperforms admission glucose in predicting coronary slow flow in patients with type 2 diabetes mellitus. Int. J. Gen. Med..

[12] Sardu C., Barbieri M., Balestrieri M.L., Siniscalchi M., Paolisso P., Calabrò P., Minicucci F., Signoriello G., Portoghese M., Mone P. (2018). Thrombus aspiration in hyperglycemic ST-elevation myocardial infarction patients: Clinical outcomes at 1-year follow-up. Cardiovasc. Diabetol..

[13] Sardu C., D’Onofrio N., Torella M., Portoghese M., Loreni F., Mureddu S., Signoriello G., Scisciola L., Barbieri M., Rizzo M.R. (2019). Pericoronary fat inflammation and major adverse cardiac events in prediabetic patients with acute myocardial infarction: Effects of metformin. Cardiovasc. Diabetol..

[14] Marfella R., Sardu C., Balestrieri M.L., Siniscalchi M., Minicucci F., Signoriello G., Calabrò P., Mauro C., Pieretti G., Coppola A. (2018). Effects of incretin treatment on cardiovascular outcomes in diabetic STEMI patients with culprit obstructive and multivessel non-obstructive coronary stenosis. Diabetol. Metab. Syndr..

[15] Akpek M., Kaya M.G., Lam Y.Y., Sahin O., Elcik D., Celik T., Ergin A., Gibson C.M. (2012). Relation of neutrophil/lymphocyte ratio to coronary flow to in-hospital major adverse cardiac events in patients with ST-elevated myocardial infarction undergoing primary coronary intervention. Am. J. Cardiol..

[16] Zhang S., Diao J., Qi C., Jin J., Li L., Gao X., Gong L., Wu W. (2018). Predictive value of neutrophil to lymphocyte ratio in patients with acute ST segment elevation myocardial infarction after percutaneous coronary intervention: A meta-analysis. BMC Cardiovasc. Disord..

[17] Zhang C., Li M., Liu L., Deng L., Xie Y., Zhong Y., Liao B., Yu L., Feng J. (2024). Systemic immune-inflammation index as a novel predictor of major adverse cardiovascular events in patients undergoing percutaneous coronary intervention: A meta-analysis of cohort studies. BMC Cardiovasc. Disord..

[18] Ul Hussain H., Kumar K.A., Zahid M., Husban Burney M., Khan Z., Asif M., Rehan S.T., Ahmad Cheema H., Swed S., Yasmin F. (2024). Neutrophil to lymphocyte ratio as a prognostic marker for cardiovascular outcomes in patients with ST-segment elevation myocardial infarction after percutaneous coronary intervention: A systematic review and meta-analysis. Medicine.

[19] Wang S., Zhang G. (2025). Association between systemic immune-inflammation index and adverse outcomes in patients with acute coronary syndrome: A meta-analysis. Angiology.

[20] Yuxiu Y., Ma X., Gao F., Liu T., Deng J., Wang Z. (2024). Combined effect of inflammation and malnutrition for long-term prognosis in patients with acute coronary syndrome undergoing percutaneous coronary intervention: A cohort study. BMC Cardiovasc. Disord..

[21] Soeters P.B., Wolfe R.R., Shenkin A. (2019). Hypoalbuminemia: Pathogenesis and clinical significance. JPEN J. Parenter. Enter. Nutr..

[22] Kalyoncuoğlu M., Katkat F., Biter H.I., Cakal S., Tosu A.R., Can M.M. (2021). Predicting one-year deaths and major adverse vascular events with the controlling nutritional status score in elderly patients with non-ST-elevated myocardial infarction undergoing percutaneous coronary intervention. J. Clin. Med..

[23] Safak O., Yildirim T., Emren V., Avci E., Argan O., Aktas Z., Yildirim S.E., Akgun D.E., Kisacik H.L. (2024). Prognostic nutritional index as a predictor of no-reflow occurrence in patients with ST-segment elevation myocardial infarction who underwent primary percutaneous coronary intervention. Angiology.

[24] Chen Q.J., Qu H.J., Li D.Z., Li X.M., Zhu J.J., Xiang Y., Li L., Ma Y.T., Yang Y.N. (2017). Prognostic nutritional index predicts clinical outcome in patients with acute ST-segment elevation myocardial infarction undergoing primary percutaneous coronary intervention. Sci. Rep..

[25] Basta G., Chatzianagnostou K., Paradossi U., Botto N., Del Turco S., Taddei A., Berti S., Mazzone A. (2016). The prognostic impact of objective nutritional indices in elderly patients with ST-elevation myocardial infarction undergoing primary coronary intervention. Int. J. Cardiol..

[26] Ignacio de Ulíbarri J., González-Madroño A., de Villar N.G.P., González P., González B., Mancha A., Rodríguez F., Fernández G. (2005). CONUT: A tool for controlling nutritional status. First validation in a hospital population. Nutr. Hosp..

[27] Saygi M., Tanalp A.C., Tezen O., Pay L., Dogan R., Uzman O., Karabay C.Y., Tanboga I.H., Kacar F.O., Karagoz A. (2024). The prognostic importance of the Naples prognostic score for in-hospital mortality in patients with ST-segment elevation myocardial infarction. Coron. Artery Dis..

[28] Şaylık F., Çınar T., Selçuk M., Akbulut T., Hayıroğlu M.İ., Tanboğa İ.H. (2024). Evaluation of Naples score for long-term mortality in patients with ST-segment elevation myocardial infarction undergoing primary percutaneous coronary intervention. Angiology.

[29] Galizia G., Lieto E., Auricchio A., Cardella F., Mabilia A., Podzemny V., Castellano P., Orditura M., Napolitano V. (2017). Naples prognostic score, based on nutritional and inflammatory status, is an independent predictor of long-term outcome in patients undergoing surgery for colorectal cancer. Dis. Colon Rectum.

[30] Topkan E., Selek U., Pehlivan B., Kucuk A., Ozturk D., Ozdemir B.S., Besen A.A., Mertsoylu H. (2023). The prognostic value of the novel global immune-nutrition-inflammation index (GINI) in stage IIIC non-small cell lung cancer patients treated with concurrent chemoradiotherapy. Cancers.

[31] Karakurt H., Karakurt S.T. (2026). The prognostic significance of the novel global immune-nutrition-inflammation index in forecasting in-hospital mortality in patients with ST-elevation myocardial infarction. Catheter. Cardiovasc. Interv..

[32] Eyiol A., Eyiol H., Yilmaz A., Sari H. (2026). Pan-immune-inflammatory value and HALP score as prognostic indicators in acute coronary syndrome patients. J. Clin. Med..

[33] Eyiol A., Ertekin B., Acar T. (2024). The association between systemic inflammatory indices and prognosis in acute coronary syndrome. Ann. Clin. Anal. Med..

