# Supplementary material for: Comparative Associations of Inflammation and Nutrition-Based Indices with No-Reflow and Mortality After Primary PCI for ST-Elevation Myocardial Infarction
Source: Biomedicines. 2026 Oct 6;14(10):2261. doi: 10.3390/biomedicines14102261 (PMC13701917; doi:10.3390/biomedicines14102261)
Supplement: Supplementary file 1 [file biomedicines-14-02261-s001.zip › biomedicines-4543450-supplementary/Supplementary_Materiald_Tables_S1-S8.pdf]

Supplementary Materials

Comparative Associations of Inflammation and Nutrition-Based Indices with No-Reflow and Mortality After Primary PCI for ST-Elevation Myocardial Infarction

Ömer Bedir [1,*], Tayfur Erdoğdu [2], Caner Türkoğlu [1], Remziye Doğan [3], Şerafettin Demir [1], İbrahim Halil Tanboğa [4] and Yusuf Ziya Şener [5,6]

S1. Supplementary Methods

Outcome-specific baseline characteristics were summarised as median [interquartile range] or n (%) and compared descriptively with the Mann–Whitney U test, Pearson chi-square test or Fisher exact test, as appropriate. These comparisons did not determine model entry.

GINI, SII and NLR were analysed on the natural-log scale. GINI, PNI, SII and NLR used three-knot restricted cubic splines with knots at the 10th, 50th and 90th percentiles. CONUT and NAPLES used a one-degree-of-freedom linear ordinal term. All reported effects compare Q3 with Q1. Overall and nonlinearity likelihood-ratio tests were reported separately.

Primary Cox and logistic models included age, sex, diabetes mellitus, hypertension, four-category Killip class and log creatinine. Post-PCI LVEF was excluded from primary models and added only in conditional sensitivity analyses. Expanded no-reflow models added total ischaemic time, infarct-related artery, pre-PCI TIMI flow, high thrombus burden, SYNTAX score and multivessel disease. Expanded early-mortality models added admission glucose, total ischaemic time and previous myocardial infarction.

Complete-case analysis was used. The primary long-term multiplicity family comprised the six Cox overall tests and used Holm correction. The 18 secondary logistic overall tests used Benjamini–Hochberg false-discovery-rate adjustment. Bootstrap coefficient-stability analyses used 500 resamples.

S2. Supplementary Results

Baseline comparisons according to outcome are shown in Supplementary Tables S1–S4. Albumin availability determined the smaller analysis samples for GINI, PNI, CONUT and NAPLES, whereas SII and NLR were available in the full cohort. Expanded no-reflow and early-mortality models generally preserved the direction of the primary estimates. Conditional adjustment for post-PCI LVEF attenuated several associations. Multiplicity-sensitive inference supported the primary long-term associations for PNI, SII and NLR. Bootstrap distributions retained the direction of most early-mortality estimates, although in-hospital models remained comparatively imprecise.

S3. Supplementary Tables

Supplementary Table S1. Baseline characteristics according to no-reflow status.

Variable	Overall	Absent	Present	P
Age, years	62 [54–70]	61 [54–70]	66 [58–76]	<0.001
Sex: Male	1881 (72.0%)	1622 (71.8%)	259 (73.4%)	0.533
Sex: Female	732 (28.0%)	638 (28.2%)	94 (26.6%)	
Diabetes mellitus	706 (27.0%)	577 (25.5%)	129 (36.5%)	<0.001
Hypertension	1280 (49.0%)	1087 (48.1%)	193 (54.7%)	0.022
Current smoking	1176 (45.0%)	1025 (45.4%)	151 (42.8%)	0.365
Dyslipidaemia	967 (37.0%)	855 (37.8%)	112 (31.7%)	0.027
Prior myocardial infarction	340 (13.0%)	304 (13.5%)	36 (10.2%)	0.091
Prior PCI	366 (14.0%)	320 (14.2%)	46 (13.0%)	0.570
Prior CABG	78 (3.0%)	72 (3.2%)	6 (1.7%)	0.127
Peripheral artery disease	183 (7.0%)	162 (7.2%)	21 (5.9%)	0.404
Killip class: 1	2198 (84.1%)	1932 (85.5%)	266 (75.4%)	<0.001
Killip class: 2	268 (10.3%)	209 (9.2%)	59 (16.7%)	
Killip class: 3	104 (4.0%)	89 (3.9%)	15 (4.2%)	
Killip class: 4	43 (1.6%)	30 (1.3%)	13 (3.7%)	
Cardiogenic shock	94 (3.6%)	73 (3.2%)	21 (5.9%)	0.011
Hypotension	246 (9.4%)	200 (8.8%)	46 (13.0%)	0.012
Systolic BP, mmHg	136 [123–147]	136 [124–147]	137 [121–160]	0.182
Heart rate, beats/min	84 [75–93]	84 [75–92]	85 [73–95]	0.916
Total ischaemic time, min	133 [76–219]	129 [74–210]	190 [107–287]	<0.001
Creatinine, mg/dL	0.84 [0.73–0.97]	0.83 [0.73–0.97]	0.86 [0.75–1.06]	<0.001
Admission glucose, mg/dL	109 [86–152]	107 [85–147]	122 [93–164]	<0.001
Haemoglobin, g/dL	14.0 [12.7–14.9]	14.1 [12.8–14.9]	13.9 [12.6–14.9]	0.641
White blood cells, 10^9/L	11.7 [9.6–13.9]	11.7 [9.6–13.8]	11.8 [10.0–14.7]	0.024
Neutrophils, 10^9/L	8.8 [6.9–10.8]	8.7 [6.8–10.8]	9.3 [7.3–11.8]	<0.001
Lymphocytes, 10^9/L	1.5 [1.0–2.1]	1.5 [1.0–2.1]	1.2 [0.8–1.8]	<0.001
Monocytes, 10^9/L	0.00 [0.00–0.00]	0.00 [0.00–0.00]	0.00 [0.00–0.00]	0.108
Platelets, 10^9/L	238 [200–284]	239 [201–285]	233 [194–278]	0.122
CRP, mg/L	7.4 [2.9–13.1]	7.2 [2.8–12.6]	8.6 [3.4–17.9]	<0.001
Albumin, g/dL	3.8 [3.6–4.1]	3.8 [3.6–4.1]	3.7 [3.4–4.0]	<0.001
Total cholesterol, mg/dL	192 [166–213]	191 [168–213]	193 [157–212]	0.268
Post-PCI LVEF, %	46 [40–52]	47 [41–53]	41 [36–47]	<0.001
Thrombus burden: 2	108 (4.1%)	103 (4.6%)	5 (1.4%)	<0.001
Thrombus burden: 3	349 (13.4%)	328 (14.5%)	21 (5.9%)	
Thrombus burden: 4	135 (5.2%)	119 (5.3%)	16 (4.5%)	
Thrombus burden: 5	2021 (77.3%)	1710 (75.7%)	311 (88.1%)	
SYNTAX score	18.6 [11.1–21.1]	18.6 [11.1–21.1]	19.1 [11.1–24.1]	0.157
Multivessel disease	1100 (42.1%)	951 (42.1%)	149 (42.2%)	0.963
GINI	2.18 [0.71–6.00]	2.06 [0.66–5.46]	3.72 [1.19–10.84]	<0.001
PNI	45.73 [41.28–50.28]	45.98 [41.73–50.73]	43.23 [39.23–48.24]	<0.001
CONUT	2 [0–3]	2 [0–3]	2 [1–4]	<0.001
SII	1365 [829–2345]	1320 [812–2231]	1781 [971–3056]	<0.001
NLR	5.67 [3.69–9.57]	5.49 [3.60–9.11]	7.94 [4.19–13.22]	<0.001
NAPLES	2 [1–2]	2 [1–2]	2 [1–3]	0.012
In-hospital death	102 (3.9%)	77 (3.4%)	25 (7.1%)	<0.001
30-day death	157 (6.0%)	121 (5.4%)	36 (10.2%)	<0.001
Long-term death	466 (17.8%)	365 (16.2%)	101 (28.6%)	<0.001

Continuous variables are median [interquartile range]; categorical variables are n (%). P values are descriptive.

Abbreviations: CABG, coronary artery bypass grafting; CRP, C-reactive protein; LVEF, left ventricular ejection fraction; PCI, percutaneous coronary intervention; SYNTAX, Synergy between Percutaneous Coronary Intervention with TAXUS and Cardiac Surgery; CONUT, controlling nutritional status; GINI, global immune-nutrition-inflammation index; NAPLES, Naples prognostic score; NLR, neutrophil-to-lymphocyte ratio; PNI, prognostic nutritional index; SII, systemic immune-inflammation index.

Supplementary Table S2. Baseline characteristics according to in-hospital mortality.

Variable	Overall	Absent	Present	P
Age, years	62 [54–70]	62 [54–70]	69 [60–80]	<0.001
Sex: Male	1881 (72.0%)	1819 (72.4%)	62 (60.8%)	0.010
Sex: Female	732 (28.0%)	692 (27.6%)	40 (39.2%)	
Diabetes mellitus	706 (27.0%)	647 (25.8%)	59 (57.8%)	<0.001
Hypertension	1280 (49.0%)	1234 (49.1%)	46 (45.1%)	0.423
Current smoking	1176 (45.0%)	1152 (45.9%)	24 (23.5%)	<0.001
Dyslipidaemia	967 (37.0%)	932 (37.1%)	35 (34.3%)	0.565
Prior myocardial infarction	340 (13.0%)	317 (12.6%)	23 (22.5%)	0.003
Prior PCI	366 (14.0%)	353 (14.1%)	13 (12.7%)	0.708
Prior CABG	78 (3.0%)	70 (2.8%)	8 (7.8%)	0.010
Peripheral artery disease	183 (7.0%)	177 (7.0%)	6 (5.9%)	0.651
Killip class: 1	2198 (84.1%)	2161 (86.1%)	37 (36.3%)	<0.001
Killip class: 2	268 (10.3%)	251 (10.0%)	17 (16.7%)	
Killip class: 3	104 (4.0%)	76 (3.0%)	28 (27.5%)	
Killip class: 4	43 (1.6%)	23 (0.9%)	20 (19.6%)	
Cardiogenic shock	94 (3.6%)	52 (2.1%)	42 (41.2%)	<0.001
Hypotension	246 (9.4%)	193 (7.7%)	53 (52.0%)	<0.001
Systolic BP, mmHg	136 [123–147]	137 [125–147]	95 [75–142]	<0.001
Heart rate, beats/min	84 [75–93]	84 [75–92]	95 [73–111]	<0.001
Total ischaemic time, min	133 [76–219]	132 [76–216]	201 [125–277]	<0.001
Creatinine, mg/dL	0.84 [0.73–0.97]	0.83 [0.73–0.96]	1.15 [0.93–1.47]	<0.001
Admission glucose, mg/dL	109 [86–152]	108 [85–147]	179 [120–272]	<0.001
Haemoglobin, g/dL	14.0 [12.7–14.9]	14.1 [12.9–14.9]	13.4 [11.6–14.5]	<0.001
White blood cells, 10^9/L	11.7 [9.6–13.9]	11.6 [9.6–13.9]	14.0 [11.4–20.4]	<0.001
Neutrophils, 10^9/L	8.8 [6.9–10.8]	8.7 [6.8–10.8]	11.0 [9.1–16.8]	<0.001
Lymphocytes, 10^9/L	1.5 [1.0–2.1]	1.5 [1.0–2.1]	1.4 [1.0–1.8]	0.029
Monocytes, 10^9/L	0.00 [0.00–0.00]	0.00 [0.00–0.00]	0.00 [0.00–0.00]	<0.001
Platelets, 10^9/L	238 [200–284]	238 [200–283]	252 [206–298]	0.160
CRP, mg/L	7.4 [2.9–13.1]	7.0 [2.8–12.7]	15.6 [7.9–26.4]	<0.001
Albumin, g/dL	3.8 [3.6–4.1]	3.8 [3.6–4.1]	3.3 [3.0–3.8]	<0.001
Total cholesterol, mg/dL	192 [166–213]	192 [167–213]	182 [149–203]	0.001
Post-PCI LVEF, %	46 [40–52]	47 [41–52]	33 [29–39]	<0.001
Thrombus burden: 2	108 (4.1%)	108 (4.3%)	0 (0.0%)	0.002
Thrombus burden: 3	349 (13.4%)	344 (13.7%)	5 (4.9%)	
Thrombus burden: 4	135 (5.2%)	131 (5.2%)	4 (3.9%)	
Thrombus burden: 5	2021 (77.3%)	1928 (76.8%)	93 (91.2%)	
SYNTAX score	18.6 [11.1–21.1]	18.6 [11.1–21.1]	23.1 [16.2–30.9]	<0.001
Multivessel disease	1100 (42.1%)	1029 (41.0%)	71 (69.6%)	<0.001
GINI	2.18 [0.71–6.00]	2.07 [0.68–5.54]	9.80 [3.88–28.55]	<0.001
PNI	45.73 [41.28–50.28]	45.78 [41.73–50.45]	39.23 [35.76–45.23]	<0.001
CONUT	2 [0–3]	2 [0–3]	4 [2–5]	<0.001
SII	1365 [829–2345]	1340 [818–2321]	2094 [1237–4335]	<0.001
NLR	5.67 [3.69–9.57]	5.52 [3.64–9.46]	8.45 [5.77–15.22]	<0.001
NAPLES	2 [1–2]	2 [1–2]	2 [2–3]	<0.001
No-reflow	353 (13.5%)	328 (13.1%)	25 (24.5%)	<0.001

Continuous variables are median [interquartile range]; categorical variables are n (%). P values are descriptive.

Abbreviations: CABG, coronary artery bypass grafting; CRP, C-reactive protein; LVEF, left ventricular ejection fraction; PCI, percutaneous coronary intervention; SYNTAX, Synergy between Percutaneous Coronary Intervention with TAXUS and Cardiac Surgery; CONUT, controlling nutritional status; GINI, global immune-nutrition-inflammation index; NAPLES, Naples prognostic score; NLR, neutrophil-to-lymphocyte ratio; PNI, prognostic nutritional index; SII, systemic immune-inflammation index.

Supplementary Table S3. Baseline characteristics according to 30-day mortality.

Variable	Overall	Absent	Present	P
Age, years	62 [54–70]	61 [54–70]	68 [59–78]	<0.001
Sex: Male	1881 (72.0%)	1783 (72.6%)	98 (62.4%)	0.006
Sex: Female	732 (28.0%)	673 (27.4%)	59 (37.6%)	
Diabetes mellitus	706 (27.0%)	629 (25.6%)	77 (49.0%)	<0.001
Hypertension	1280 (49.0%)	1213 (49.4%)	67 (42.7%)	0.103
Current smoking	1176 (45.0%)	1131 (46.1%)	45 (28.7%)	<0.001
Dyslipidaemia	967 (37.0%)	909 (37.0%)	58 (36.9%)	0.986
Prior myocardial infarction	340 (13.0%)	307 (12.5%)	33 (21.0%)	0.002
Prior PCI	366 (14.0%)	342 (13.9%)	24 (15.3%)	0.634
Prior CABG	78 (3.0%)	69 (2.8%)	9 (5.7%)	0.049
Peripheral artery disease	183 (7.0%)	175 (7.1%)	8 (5.1%)	0.334
Killip class: 1	2198 (84.1%)	2114 (86.1%)	84 (53.5%)	<0.001
Killip class: 2	268 (10.3%)	249 (10.1%)	19 (12.1%)	
Killip class: 3	104 (4.0%)	71 (2.9%)	33 (21.0%)	
Killip class: 4	43 (1.6%)	22 (0.9%)	21 (13.4%)	
Cardiogenic shock	94 (3.6%)	48 (2.0%)	46 (29.3%)	<0.001
Hypotension	246 (9.4%)	186 (7.6%)	60 (38.2%)	<0.001
Systolic BP, mmHg	136 [123–147]	137 [125–147]	127 [77–145]	<0.001
Heart rate, beats/min	84 [75–93]	84 [75–92]	89 [74–109]	<0.001
Total ischaemic time, min	133 [76–219]	131 [74–215]	190 [107–277]	<0.001
Creatinine, mg/dL	0.84 [0.73–0.97]	0.83 [0.73–0.95]	1.05 [0.88–1.45]	<0.001
Admission glucose, mg/dL	109 [86–152]	108 [85–146]	146 [101–247]	<0.001
Haemoglobin, g/dL	14.0 [12.7–14.9]	14.1 [12.9–14.9]	13.7 [11.4–14.9]	0.002
White blood cells, 10^9/L	11.7 [9.6–13.9]	11.6 [9.6–13.9]	13.2 [10.9–17.3]	<0.001
Neutrophils, 10^9/L	8.8 [6.9–10.8]	8.7 [6.8–10.8]	10.4 [8.5–13.9]	<0.001
Lymphocytes, 10^9/L	1.5 [1.0–2.1]	1.5 [1.0–2.1]	1.3 [0.9–1.8]	<0.001
Monocytes, 10^9/L	0.00 [0.00–0.00]	0.00 [0.00–0.00]	0.00 [0.00–0.00]	0.006
Platelets, 10^9/L	238 [200–284]	238 [200–283]	244 [203–296]	0.288
CRP, mg/L	7.4 [2.9–13.1]	6.8 [2.7–12.5]	15.0 [7.0–25.8]	<0.001
Albumin, g/dL	3.8 [3.6–4.1]	3.8 [3.6–4.1]	3.5 [3.1–3.9]	<0.001
Total cholesterol, mg/dL	192 [166–213]	192 [167–214]	184 [157–208]	0.004
Post-PCI LVEF, %	46 [40–52]	47 [41–52]	34 [29–44]	<0.001
Thrombus burden: 2	108 (4.1%)	107 (4.4%)	1 (0.6%)	0.003
Thrombus burden: 3	349 (13.4%)	338 (13.8%)	11 (7.0%)	
Thrombus burden: 4	135 (5.2%)	130 (5.3%)	5 (3.2%)	
Thrombus burden: 5	2021 (77.3%)	1881 (76.6%)	140 (89.2%)	
SYNTAX score	18.6 [11.1–21.1]	18.6 [11.1–21.1]	21.1 [17.6–28.1]	<0.001
Multivessel disease	1100 (42.1%)	1004 (40.9%)	96 (61.1%)	<0.001
GINI	2.18 [0.71–6.00]	2.06 [0.67–5.48]	9.07 [2.79–27.83]	<0.001
PNI	45.73 [41.28–50.28]	45.78 [41.73–50.46]	40.70 [37.48–46.21]	<0.001
CONUT	2 [0–3]	2 [0–3]	3 [2–5]	<0.001
SII	1365 [829–2345]	1334 [818–2298]	2055 [1067–4335]	<0.001
NLR	5.67 [3.69–9.57]	5.51 [3.64–9.34]	8.41 [4.84–15.66]	<0.001
NAPLES	2 [1–2]	2 [1–2]	2 [2–3]	<0.001
No-reflow	353 (13.5%)	317 (12.9%)	36 (22.9%)	<0.001

Continuous variables are median [interquartile range]; categorical variables are n (%). P values are descriptive.

Abbreviations: CABG, coronary artery bypass grafting; CRP, C-reactive protein; LVEF, left ventricular ejection fraction; PCI, percutaneous coronary intervention; SYNTAX, Synergy between Percutaneous Coronary Intervention with TAXUS and Cardiac Surgery; CONUT, controlling nutritional status; GINI, global immune-nutrition-inflammation index; NAPLES, Naples prognostic score; NLR, neutrophil-to-lymphocyte ratio; PNI, prognostic nutritional index; SII, systemic immune-inflammation index.

Supplementary Table S4. Baseline characteristics according to long-term mortality status.

Variable	Overall	Absent	Present	P
Age, years	62 [54–70]	61 [53–69]	68 [59–79]	<0.001
Sex: Male	1881 (72.0%)	1592 (74.1%)	289 (62.0%)	<0.001
Sex: Female	732 (28.0%)	555 (25.9%)	177 (38.0%)	
Diabetes mellitus	706 (27.0%)	522 (24.3%)	184 (39.5%)	<0.001
Hypertension	1280 (49.0%)	1035 (48.2%)	245 (52.6%)	0.087
Current smoking	1176 (45.0%)	1002 (46.7%)	174 (37.3%)	<0.001
Dyslipidaemia	967 (37.0%)	808 (37.6%)	159 (34.1%)	0.154
Prior myocardial infarction	340 (13.0%)	256 (11.9%)	84 (18.0%)	<0.001
Prior PCI	366 (14.0%)	296 (13.8%)	70 (15.0%)	0.486
Prior CABG	78 (3.0%)	65 (3.0%)	13 (2.8%)	0.785
Peripheral artery disease	183 (7.0%)	145 (6.8%)	38 (8.2%)	0.283
Killip class: 1	2198 (84.1%)	1878 (87.5%)	320 (68.7%)	<0.001
Killip class: 2	268 (10.3%)	197 (9.2%)	71 (15.2%)	
Killip class: 3	104 (4.0%)	56 (2.6%)	48 (10.3%)	
Killip class: 4	43 (1.6%)	16 (0.7%)	27 (5.8%)	
Cardiogenic shock	94 (3.6%)	34 (1.6%)	60 (12.9%)	<0.001
Hypotension	246 (9.4%)	152 (7.1%)	94 (20.2%)	<0.001
Systolic BP, mmHg	136 [123–147]	136 [124–147]	137 [117–152]	0.951
Heart rate, beats/min	84 [75–93]	84 [75–92]	87 [75–98]	<0.001
Total ischaemic time, min	133 [76–219]	128 [73–212]	167 [99–264]	<0.001
Creatinine, mg/dL	0.84 [0.73–0.97]	0.82 [0.72–0.95]	0.92 [0.77–1.17]	<0.001
Admission glucose, mg/dL	109 [86–152]	107 [84–141]	125 [96–189]	<0.001
Haemoglobin, g/dL	14.0 [12.7–14.9]	14.1 [13.1–14.9]	13.6 [11.3–14.8]	<0.001
White blood cells, 10^9/L	11.7 [9.6–13.9]	11.6 [9.5–13.7]	12.6 [10.3–15.2]	<0.001
Neutrophils, 10^9/L	8.8 [6.9–10.8]	8.6 [6.7–10.6]	9.9 [7.8–12.4]	<0.001
Lymphocytes, 10^9/L	1.5 [1.0–2.1]	1.6 [1.0–2.2]	1.2 [0.8–1.8]	<0.001
Monocytes, 10^9/L	0.00 [0.00–0.00]	0.00 [0.00–0.00]	0.00 [0.00–0.00]	0.035
Platelets, 10^9/L	238 [200–284]	238 [200–283]	243 [205–288]	0.235
CRP, mg/L	7.4 [2.9–13.1]	6.4 [2.5–12.1]	10.7 [5.2–20.6]	<0.001
Albumin, g/dL	3.8 [3.6–4.1]	3.8 [3.6–4.1]	3.7 [3.2–4.0]	<0.001
Total cholesterol, mg/dL	192 [166–213]	193 [168–214]	186 [158–212]	0.003
Post-PCI LVEF, %	46 [40–52]	47 [42–53]	38 [33–46]	<0.001
Thrombus burden: 2	108 (4.1%)	98 (4.6%)	10 (2.1%)	0.012
Thrombus burden: 3	349 (13.4%)	296 (13.8%)	53 (11.4%)	
Thrombus burden: 4	135 (5.2%)	117 (5.4%)	18 (3.9%)	
Thrombus burden: 5	2021 (77.3%)	1636 (76.2%)	385 (82.6%)	
SYNTAX score	18.6 [11.1–21.1]	17.6 [11.1–21.1]	20.1 [15.6–25.9]	<0.001
Multivessel disease	1100 (42.1%)	851 (39.6%)	249 (53.4%)	<0.001
GINI	2.18 [0.71–6.00]	1.89 [0.62–4.90]	5.94 [1.68–16.00]	<0.001
PNI	45.73 [41.28–50.28]	46.23 [42.23–50.76]	42.23 [37.78–47.23]	<0.001
CONUT	2 [0–3]	2 [0–3]	3 [1–5]	<0.001
SII	1365 [829–2345]	1273 [797–2153]	1975 [1030–3487]	<0.001
NLR	5.67 [3.69–9.57]	5.31 [3.55–8.85]	8.24 [4.81–13.21]	<0.001
NAPLES	2 [1–2]	2 [1–2]	2 [2–3]	<0.001

Continuous variables are median [interquartile range]; categorical variables are n (%). P values are descriptive.

Abbreviations: CABG, coronary artery bypass grafting; CRP, C-reactive protein; LVEF, left ventricular ejection fraction; PCI, percutaneous coronary intervention; SYNTAX, Synergy between Percutaneous Coronary Intervention with TAXUS and Cardiac Surgery; CONUT, controlling nutritional status; GINI, global immune-nutrition-inflammation index; NAPLES, Naples prognostic score; NLR, neutrophil-to-lymphocyte ratio; PNI, prognostic nutritional index; SII, systemic immune-inflammation index.

Supplementary Table S5A. Score distributions, transformations and knot locations.

Index	Available n	Q1	Median	Q3	Model specification	Knot 10	Knot 50	Knot 90
GINI	1932	0.71	2.18	6.00	natural log + 3-knot RCS	-1.529	0.779	2.667
PNI	1935	41.28	45.73	50.28	3-knot RCS on original scale	37.780	45.730	55.780
CONUT	1935	0	2	3	linear ordinal	—	—	—
SII	2613	829	1365	2345	natural log + 3-knot RCS	6.304	7.219	8.246
NLR	2613	3.69	5.67	9.57	natural log + 3-knot RCS	0.884	1.735	2.751
NAPLES	1934	1	2	2	linear ordinal	—	—	—

Knots for log-transformed indices are shown on the model scale. Effects were reported on the original clinical scale as Q3 versus Q1.

Abbreviations: Q1, first quartile (25th percentile); Q3, third quartile (75th percentile); RCS, restricted cubic spline; CONUT, controlling nutritional status; GINI, global immune-nutrition-inflammation index; NAPLES, Naples prognostic score; NLR, neutrophil-to-lymphocyte ratio; PNI, prognostic nutritional index; SII, systemic immune-inflammation index.

Supplementary Table S5B. Knot locations for continuous clinical covariates.

Variable	Knot 10	Knot 50	Knot 90
Age, years	48	62	79
Log creatinine	-0.46	-0.17	0.16
Post-PCI LVEF, %	34	46	54

Age, log creatinine and post-PCI LVEF were modelled flexibly as indicated.

Abbreviations: LVEF, left ventricular ejection fraction; PCI, percutaneous coronary intervention.

Supplementary Table S6. Expanded early-mortality models.

Endpoint	Index	Contrast (Q3 vs Q1)	N / events	Adjusted effect (95% CI)	Overall P	P for nonlinearity	Model df	Events / parameter
In-hospital mortality	GINI	6.00 vs 0.71	1932 / 75	2.60 (1.35–5.04)	<0.001	0.673	15	5
In-hospital mortality	PNI	50.28 vs 41.28	1935 / 75	0.49 (0.33–0.73)	<0.001	0.388	15	5
In-hospital mortality	CONUT	3 vs 0	1935 / 75	1.68 (1.17–2.43)	0.006	—	14	5.4
In-hospital mortality	SII	2345 vs 829	2613 / 102	1.38 (0.95–2.02)	0.024	0.883	15	6.8
In-hospital mortality	NLR	9.57 vs 3.69	2613 / 102	1.62 (1.09–2.41)	0.014	0.349	15	6.8
In-hospital mortality	NAPLES	2 vs 1	1934 / 75	1.76 (1.21–2.56)	0.002	—	14	5.4
30-day mortality	GINI	6.00 vs 0.71	1932 / 112	1.90 (1.39–2.60)	<0.001	0.114	15	7.5
30-day mortality	PNI	50.28 vs 41.28	1935 / 112	0.59 (0.45–0.77)	<0.001	0.254	15	7.5
30-day mortality	CONUT	3 vs 0	1935 / 112	1.74 (1.28–2.36)	<0.001	—	14	8
30-day mortality	SII	2345 vs 829	2613 / 157	1.28 (0.98–1.68)	<0.001	0.064	15	10.5
30-day mortality	NLR	9.57 vs 3.69	2613 / 157	1.48 (1.12–1.95)	<0.001	0.660	15	10.5
30-day mortality	NAPLES	2 vs 1	1934 / 112	1.34 (1.01–1.80)	0.043	—	14	8

Expanded models included the core clinical variables plus admission glucose, total ischaemic time and previous myocardial infarction.

Abbreviations: CI, confidence interval; df, degrees of freedom; N / events, number of patients in the model and number of events; Q1, first quartile (25th percentile); Q3, third quartile (75th percentile); CONUT, controlling nutritional status; GINI, global immune-nutrition-inflammation index; NAPLES, Naples prognostic score; NLR, neutrophil-to-lymphocyte ratio; PNI, prognostic nutritional index; SII, systemic immune-inflammation index.

Supplementary Table S7. Multiplicity-sensitive analyses.

Endpoint	Index	Raw overall P	Method	Adjusted P or q
Long-term mortality	GINI	0.113	Holm	0.184
Long-term mortality	PNI	0.001	Holm	0.007
Long-term mortality	CONUT	0.042	Holm	0.127
Long-term mortality	SII	0.008	Holm	0.031
Long-term mortality	NLR	0.002	Holm	0.010
Long-term mortality	NAPLES	0.092	Holm	0.184
No-reflow	GINI	<0.001	Benjamini-Hochberg	<0.001
In-hospital mortality	GINI	<0.001	Benjamini-Hochberg	<0.001
30-day mortality	GINI	<0.001	Benjamini-Hochberg	<0.001
No-reflow	PNI	0.018	Benjamini-Hochberg	0.021
In-hospital mortality	PNI	<0.001	Benjamini-Hochberg	<0.001
30-day mortality	PNI	<0.001	Benjamini-Hochberg	<0.001
No-reflow	CONUT	0.010	Benjamini-Hochberg	0.013
In-hospital mortality	CONUT	0.005	Benjamini-Hochberg	0.007
30-day mortality	CONUT	<0.001	Benjamini-Hochberg	<0.001
No-reflow	SII	<0.001	Benjamini-Hochberg	<0.001
In-hospital mortality	SII	0.015	Benjamini-Hochberg	0.019
30-day mortality	SII	<0.001	Benjamini-Hochberg	<0.001
No-reflow	NLR	<0.001	Benjamini-Hochberg	<0.001
In-hospital mortality	NLR	0.007	Benjamini-Hochberg	0.009
30-day mortality	NLR	<0.001	Benjamini-Hochberg	<0.001
No-reflow	NAPLES	0.567	Benjamini-Hochberg	0.567
In-hospital mortality	NAPLES	0.002	Benjamini-Hochberg	0.003
30-day mortality	NAPLES	0.034	Benjamini-Hochberg	0.036

Holm correction was used for the six primary Cox tests; Benjamini–Hochberg correction was used for the 18 secondary logistic tests.

Abbreviations: q, false-discovery-rate-adjusted P value; CONUT, controlling nutritional status; GINI, global immune-nutrition-inflammation index; NAPLES, Naples prognostic score; NLR, neutrophil-to-lymphocyte ratio; PNI, prognostic nutritional index; SII, systemic immune-inflammation index.

Supplementary Table S8. Bootstrap coefficient-stability analysis.

Endpoint	Index	N / events	Original OR	Bootstrap median OR	Bootstrap 95% interval	Same direction, %
In-hospital mortality	GINI	1932 / 75	2.77	2.91	1.92–8.12	100
In-hospital mortality	PNI	1935 / 75	0.50	0.48	0.28–0.70	100
In-hospital mortality	CONUT	1935 / 75	1.68	1.69	1.20–2.48	100
In-hospital mortality	SII	2613 / 102	1.44	1.47	1.04–2.31	98.2
In-hospital mortality	NLR	2613 / 102	1.69	1.69	1.16–2.81	99.8
In-hospital mortality	NAPLES	1934 / 75	1.77	1.78	1.24–2.67	99.8
30-day mortality	GINI	1932 / 112	2.00	2.03	1.56–2.84	100
30-day mortality	PNI	1935 / 112	0.58	0.58	0.42–0.75	100
30-day mortality	CONUT	1935 / 112	1.75	1.75	1.37–2.31	100
30-day mortality	SII	2613 / 157	1.35	1.35	1.06–1.83	99.4
30-day mortality	NLR	2613 / 157	1.56	1.55	1.20–2.12	100
30-day mortality	NAPLES	1934 / 112	1.36	1.36	0.99–1.88	96.2

Odds-ratio summaries are exponentiated from the bootstrap log-odds contrasts. Five hundred patient-level resamples were requested.

Abbreviations: N / events, number of patients in the model and number of events; OR, odds ratio; CONUT, controlling nutritional status; GINI, global immune-nutrition-inflammation index; NAPLES, Naples prognostic score; NLR, neutrophil-to-lymphocyte ratio; PNI, prognostic nutritional index; SII, systemic immune-inflammation index.
